# Personality traits and transition to psychosis one year after the first assessment

**DOI:** 10.3389/fpsyg.2023.1096626

**Published:** 2023-01-19

**Authors:** Francesca De Salve, Chiara Rossi, Cesare Cavalera, Lara Malvini, Simona Barbera, Sofia Tagliabue, Mauro Percudani, Osmano Oasi

**Affiliations:** ^1^Department of Psychology, Catholic University of Milan, Milan, Italy; ^2^Department of Mental Health and Addiction Services, Niguarda Hospital, Milan, Italy

**Keywords:** at-risk mental states, ultra high risk, psychosis, personality traits, PID-5, social and occupational functioning, detachment and disinhibition

## Abstract

**Introduction:**

Several studies have identified ultra-high-risk criteria that may characterize an at-risk mental state and predict the transition of psychotic evolution. Personality traits may play a crucial role in this process.

**Aims:**

The current study aims to: (a) explore the evolution of an initial diagnosis over 12 months; (b) assess differences in social and occupational functioning; (c) identify common (trans-diagnostic) personality traits of psychotic risk.

**Methods:**

The sample includes 97 (44 males and 53 females) young adults. They completed an assessment that consists of socio-demographic data, the Social and Occupational Functioning Scale, the Early Recognition Inventory-retrospective assessment onset of schizophrenia, and the Personality Inventory for DSM-5 (PID-5). According to the tests’ assessment, the sample was divided into three different groups: Ultra-High Risk (UHR), At-Risk, and Not at risk. One year after the first evaluation, psychiatrists administered the QuickSCID-5 to verify the diagnostic trajectories of the sample.

**Results:**

Overall, the most prevalent category diagnoses were anxiety/depression, personality disorders, and psychosis. Specifically, the most common diagnosis in the UHR group was psychosis. Moreover, in the UHR group, the social and occupational functioning score was the lowest. In terms of differences in PID-5 personality traits, the At-risk and UHR groups scored highest in detachment and disinhibition. No statistically significant differences were found between the groups for negative affectivity, antagonism, and psychoticism traits.

**Conclusion:**

Results obtained by the current study should be considered an attempt to better understand the diagnostic trajectories and trans-diagnostic personality traits in a group of young help-seekers, specifically in UHR. Findings highlight both the importance of diagnosis and personality traits evaluation to customize a specific intervention based on the level of psychotic risk. Clinical suggestions are reported.

## 1. Introduction

Different psychotic manifestations can be read according to a developmental perspective; indeed, in the natural history of the psychological disease, it is frequently observed the presence of different phases, prodromal, acute, and chronic (Yung et al., 2003). The prodromal phase has an average duration of between 1 and 5 years and is often associated with high psychosocial impairment and disability (Loebel et al., 1992; Beiser et al., 1993; Häfner et al., 1993). Prodromal states are characterized by non-specific symptoms of different nature, including restlessness, concentration, social and cognitive difficulties, fear, low self-esteem, social withdrawal, poor school or work performance, worsening quality of life, anxiety, sleep disorders, personality, and mood changes, and attenuated psychotic symptoms (Yung et al., 2007). Some studies have shown that very often the decrease in cognitive performance and impairment of social functioning anticipate the actual onset of the disease by up to several years; these dysfunctions act as both maintenance factors and transition markers (Tarbox et al., 2014; Nelson et al., 2021). Whereas the acute phase corresponds to the psychotic onset, the chronic one is characterized by the continuative presence of positive, and negative, cognitive symptoms and functional disabilities (Yung et al., 2003).

The original At-Risk Mental State (ARMS) construct – also referred to as the Clinical High-Risk State for psychosis (CHR-P) – was introduced in 1996 to identify young people at increased risk of having a first psychotic episode and thus developing an overt psychotic disorder (Yung et al., 2005; Fusar-Poli, 2017). A strategy has been adopted to identify young people with ARMS. This strategy is based on identifying risk factors for psychotic disorders, i.e., trait factors (genetic, schizotypal personality disorder, or a family history of psychosis), state factors (such as mental distress and deterioration in functioning), specific symptomatology presenting prior to onset and age between 15 and 25 years (Nelson, 2014).

Ultra-High Risk (UHR) persons present with ARMS and a decline or persistently low social and occupational functioning (Nelson, 2014). The UHR criteria allow three groups to be identified in turn: the vulnerable group – schizotypal disorder and/or first-degree relative with a psychotic disorder, Attenuated Psychotic Symptoms (APS) – the presence of brief, limited, and intermittent psychotic symptoms, Brief Limited Intermittent Psychotic Symptoms (BLIPS) – positive psychotic symptoms that did not last more than a week and disappeared without treatment. Young people who meet the UHR criteria have subthreshold symptoms for a psychotic episode, however, not all of them will necessarily have a transition to psychosis. We speak of a first psychotic episode when symptoms go from subthreshold to full-blown (both in intensity and frequency) (Nelson, 2014).

The concept of At-Risk Mental State becomes crucial in clinical practice, especially from a preventive perspective (Lin et al., 2012). Recognizing in advance certain signs that could lead to the development of a psychotic pathology, allows early intervention to reduce the likelihood of its onset and the related social consequences (Wölwer, 2018). Literature shows the transition rate to full-blow psychosis – among high-risk patients – is between 35 and 41% (Yung et al., 2003, 2004; Radua et al., 2018; Oliver et al., 2020).

The early intervention treatment aims to prevent the psychotic onset, when possible, and reduce the damage when primary prevention is no longer applicable. Whether the patient is already in the care of the service, it is possible to reduce the DUP (Duration of Untreated Psychosis) and improve the prognosis regarding general functioning. (Pelizza et al., 2022) showed that a psychotherapeutic and/or psychoeducational intervention, during the risk phase, significantly reduces the probability of onset in the following 12 months. Notwithstanding, the intention to create a stage model is somewhat limited by the lack of knowledge of the factors that modulate the level of risk in these individuals.

Symptoms represent an epiphenomenon of an underlying etiopathology. The identification of associated states and outcomes is entirely symptom-based. Indeed, the general pattern underlying the development of psychosis involves the culmination of genetic and environmental factors that may increase (risk factors) or decrease (protective factors) the likelihood of developing psychosis, as well as the interplay between them (Insel, 2010; Radua et al., 2018). In this regard, one strand of scientific research is trying to identify, personality traits that can predict both the level of risk and the likelihood of developing the disorder (Drvaric et al., 2018; Meliante et al., 2021). Fusar-Poli et al. (2014) found anxiety and depression play a central role in psychotic transition whereas, empirical findings on the role of personality are still controversial, and scarcely comprehensive. A recent meta-analysis (Boldrini et al., 2019) showed that 39.4% of high-risk patients have comorbidity with personality disorders and the most common diagnoses in this clinical population are schizotypal and borderline.

Existing studies indicate that schizotypy assessed in “at risk” individuals can be considered a predictor of transition from CHR-P to psychosis. This approach, however, is burdened by biases stemming from a possible overlap between current psychopathology and schizotypal features (Kotlicka-Antczak et al., 2019). The schizotypy construct reflects a phenotypic expression of vulnerability to schizophrenia and it can be conceived as part of a normal personality, which may nevertheless form a background for the development of psychotic illness (Claridge and Beech, 1995). Today, schizophrenia is in fact considered a neurodevelopmental disorder (McMillan et al., 2009; Murray et al., 2022). Clinically, the pathological process may progress to full-blown illness through the development of subtle abnormalities in cognitive and social functioning and a distinct pre-psychotic phase, currently known as clinical high-risk psychosis (Fusar-Poli, 2017). However, following a meta-analysis of the material in the literature, Debbané et al. (2015) concluded that the positive dimension of schizotypy presents little clinically significant predictive value for the transition from CHR-P to psychosis. Physical anhedonia appeared the most predictive indicator of conversion while other studies show no link between the baseline level of schizotypy and transition (Debbané et al., 2015).

Specifically, with borderline personality disorder, there is an overlap in deficits in interpersonal relationships and detachment, while with the latter there is vulnerability to disinhibition, negative affectivity, emotional dysregulation, anxiety, and depression (Smith et al., 2009; Woods et al., 2009; Debbané et al., 2015). Instead, other studies have focused on identifying the most prevalent personality traits in high-risk patients. Studies based on Personality Inventory for DSM-5 (PID-5; Fossati et al., 2013) highlighted an additional element to distinguish high-risk patients which are detachment, disinhibition, negative affectivity, and psychoticism (Drvaric et al., 2018; Shi et al., 2018; Meliante et al., 2021). Drvaric et al. (2018) have shown that patients at higher risk for psychosis score higher on two of the five AMPD trait domains – negative affectivity and detachment – than patients at lower risk. Furthermore, the authors argue that maladaptive AMPD personality traits may be a potential risk factor for conversion to psychosis. Meliante et al. (2021) have highlighted that higher scores in detachment and psychoticism may distinguish people who are more vulnerable to psychosis or who already have manifest psychosis from those who do not have a psychotic predisposition.

For this reason, the current study has three main aims: (a) exploring the evolution of the initial diagnosis over 12 months among patients recruited and assigned to three groups (Not at risk, At risk, Ultra High Risk) (b) assessing differences in social and occupational functioning between the groups (c) identifying common (trans-diagnostic) personality traits of psychotic risk groups.

## 2. Materials and methods

### 2.1. Participants

Of an initial sample of 110 individuals who in 2019 referred to the Youth Mental Health Service for Early Intervention at Niguarda Hospital in Milan (Italy), 97 participants were included in the study. They were 44 males and 53 females with an average age of 20.5 years (SD = 2.17).

The following exclusion criteria were applied: (a) incomplete personality assessment, (b) diagnosis of intellectual disability or autism, (c) psychotic onset, and (d) dropouts after 1 year from the first consultation.

Participants completed the routine Programma 2000 assessment which is a youth mental health early intervention service implemented at Niguarda Hospital in Milan, Italy. The metropolitan area served by the program includes about 350,000 inhabitants. The project integrates the management of chronic psychotic disorders with prevention services that promote health and recovery. Patients access this service through spontaneous help-seeking or institution-mediated pathways (e.g., primary care, district mental health services, school counseling, and emergency rooms). Programma 2000 offers an individualized and customizable intervention package that includes cognitive-behavioral psychotherapy, psychoeducational and motivational sessions, family support, and therapeutic group activities (e.g., anxiety management, assertive and problem-solving training, etc., etc.).

### 2.2. Procedures

All the study participants gave their informed consent after being properly informed. The study has been authorized by the Niguarda Hospital in Milan’s Ethical Committee (Protocol 305–19,052,021) and complies with the 1964 Declaration of Helsinki’s Principles and any later amendments (World Medical Association, 2013).

The assessment proposed in Programma2000 is a key element in tailoring the intervention package. It consists of socio-demographic data, the Social and Occupational Functioning Assessment, the Early Recognition Inventory-retrospective assessment onset of schizophrenia, and the Personality Inventory for DSM-5. One year after the first assessment, psychiatrists administered the QuickSCID-5 to assess the diagnostic trajectories of the patients taken into care.

### 2.3. Measures

#### 2.3.1. Quick structured clinical interview for DSM-5

The instrument is a structured interview created for making the major DSM-5 diagnoses. The QuickSCID-5 consists of 10 independent modules: Module A (episodes and mood disorders), Module B (screening for psychotic symptoms), Module C (substance use disorders), Module D (anxiety disorders), Module E (obsessive–compulsive disorder), Module F (attention-deficit/hyperactivity disorder). Module I (screening questions for other disorders), and Module J (questions for exclusion of mental disorders due to other medical conditions). Due to the modular nature, only the modules of interest can be administered (Somma et al., 2020).

#### 2.3.2. Social and occupational functioning assessment

The SOFAS is a clinician-report tool that measures functioning at the time of the assessment. It does not directly depend on how severe the psychological symptoms are, but it primarily concentrates on the person’s level of social and occupational functioning (Morosini et al., 2000). Precisely, SOFAS is structured as a comprehensive evaluation of the social and occupational functionality of a patient, rating from “superior functioning in a wide range of activities” (=100) to “inability to function in almost all areas” (<30).

#### 2.3.3. Early recognition inventory-retrospective assessment onset of schizophrenia (checklist)

The Checklist ERIraos (Maurer et al., 2018) is a semi-structured interview that aims to identify early signs of mental illness evaluating the perceived psychopathological changes and the family history of the subject. It combines the non-specific distress symptoms that may accompany the schizophrenia prodromes (such as social withdrawal and depression, persecutory thoughts, loss of sense of reality, and hallucinations indicating an elevated risk of psychotic development), into a single list of 17 items (Meneghelli et al., 2014). A score ≥12 necessitates a referral to the Early Intervention Center for additional investigation.

#### 2.3.4. Personality inventory for DSM-5

The Personality Inventory for DSM-5 (PID-5) has been created to assess the pathological personality traits of Criteria B in section III of the DSM-5 from a dimensional and inferential-contextual point of view. It is a self-report questionnaire composed of 220 items with a Likert scale (from 0 to 3). PID-5 is structured on 25 facets gathered into five main domains of personality traits: detachment, disinhibition, negative affectivity, antagonism, and psychoticism (Fossati et al., 2013).

### 2.4. Statistical analysis

According to the aims, the sample was divided into three different groups based on Programma 2000 assessment and labeled as follows: (1) Ultra-High Risk (UHR): consisting of patients who have exceeded the Checklist cut-off with a score >12. They specifically resulted to be positive for the last four items of the Checklist (which refers to frankly psychotic symptoms) and presented the risk factors for UHR diagnosis (family history of schizophrenia, a schizotypal personality disorder, the presence of emerging or worsening attenuated positive symptoms, deterioration of social and occupational functioning); (2) At Risk: consisting of patients who have exceeded the cut-offs for the Checklist but presented neither positivity to the last four items of the Checklist nor risk factors for the UHR diagnosis; and (3) Not at risk: consisting of all the patients who did not exceed the cut-offs of the assessment’s scales for at-risk mental states.

Analyses of skewness and kurtosis were performed to assess the sample’s distribution’s normality and each variable’s results within the acceptable range of ±2 (Podsakoff et al., 2003).

Firstly, Pearson’s Chi-Square Test was conducted to investigate the development of psychotic risk in the sample exploring the evolution of the initial diagnosis over 12 months among patients recruited and assigned to three groups (Not at risk, At Risk, Ultra High Risk). Differences in social and occupational functioning (considering SOFAS scores), among the groups, were then explored by conducting a One-way ANOVA. Finally, a MANOVA was used to investigate common trans-diagnostic personality traits (PID-5) of psychotic risk groups. Both ANOVA and MANOVA Bonferroni multiple comparison tests were led. Analyses were performed using SPSS 25.0 statistical software.

## 3. Results

The sample, aged 18–25 years with a mean age of 20.5 (SD = 2.17), was composed of 45.36% (=44) of males and 54.63% (=53) of females. Participants’ demographic information is reported in Table 1 including habits in misusing alcohol and substances. Most of the sample had previous contact with Child Neuropsychiatry (=45) and familiarity with psychotic disorder (=46).

**Table 1:** Socio-demographics characteristics of the sample.

Variables		Group	*N* (%)
Age			97 (100%)
M	20.5		
(SD)	(2.17)		
Gender		Male	44 (45.36%)
		Female	53 (54.63%)
Occupation		None	22 (22.68%)
		Student	6 (6.18%)
		Worker	67 (68.04%)
Misuse of alcohol		None	77 (79.38%)
		Yes	13 (13.40)
		Suspected	7 (7.22)
Misuse of substances		None	71 (73.19%)
		Yes	24 (24.74%)
		Suspected	2 (2.06%)
Previous contacts with child neuropsychiatry		Yes	45 (46.49%)
		None	52 (53.51%)
Familiarity with psychic disorder		Yes	46 (47.42%)
		None	51 (52.58%)

According to the SCID-5, the sample was divided into eight macro diagnostic categories: psychosis (=15), personality disorders (=32), bipolar disorder (=5), anxiety/depression (=27), substance/alcohol use disorders (=1), eating disorders (=2), trauma and stress-related disorders (=13), and dissociative identity disorder (=2). Pearson’s Chi-square test was performed to analyze the evolution of the initial diagnosis over 12 months. As shown in Table 2, results revealed a significant association between the eight categories after 1 year (*χ*^2^ = 45.04; *p* = 0.000).

**Table 2:** Diagnostic trajectories after 1 year from the evaluation of risk for psychosis.

Variables	Not at risk	At risk	High risk	Total	*p*-Value
*N*	21	44	32	97	
%	21.65%	45.36%	32.98%	100%	
**Primary diagnosis after 1 year**					0.000^a^
*Psychosis*	0	2	13	15	
% On total sample	0%	2.1%	13.4%	15.5%	
% On group of risk	0%	4.5%	40.6%		
*Personality disorders*	7	11	14	32	
% On total sample	7.2%	11.34%	14.43%	32.97%	
% On group of risk	33.3%	25%	43.7%	100%	
*Bipolar disorders*	0	2	3	5	
% On the total sample	0%	2.1%	3.1%	5.1%	
% On the group of risk	0%	4.5%	9.4%		
*Anxiety/Depression*	10	15	2	27	
% On total sample	10.3%	15.5%	2.06%	27.8%	
% On group of risk	47.6%	34.1%	6.3%		
*Substance/alcohol use disorders*	0	1	0	1	
% On total sample	0%	1.0%	0%	1.0%	
% On group of risk	0%	2.3%	0%		
*Eating disorders*	0	2	0	2	
% On total sample	0%	2.1%	0%	2.1%	
% On group of risk	0%	4.5%	0%		
*Trauma and stress related disorders*	3	10	0	13	
% On total sample	3,1%	10.3%	0%	13.4%	
% On group of risk	14.3%	27.7%	0%		
*Dissociative identity disorder*	1	1	0	2	
% On total sample	1.0%	1.0%	0%	2.1%	
% On group of risk	4.7%	2.27%	0%		

a Pearson’s Chi-square test.

Participants were then analyzed for their levels of social and occupational functioning. Overall, the sample presented a moderate score for SOFAS (M = 60, SD = 13.43) with a minimum score of 20 and a maximum of 85. Differences in social and occupational functioning among the three groups (Not at risk, At Risk, Ultra High Risk) were explored with a one-way ANOVA. The results (*F*(2, 94) = 9.068; *p* < 0.001) and the Bonferroni Post-Hoc (Table 3) showed higher levels of social and occupational functioning in the Not at-risk group (M = 63.86) compared to the Ultra High Risk (M = 52.34) for *p* = 0.004, and At Risk (M = 63.72) groups for *p* = 0.000.

**Table 3:** Differences in individual characteristics.

Variables	Not at risk (0)	At risk (1)	High risk (2)	Total	*p*-Value	*Post hoc*
*N*	21	44	32	97		
*Gender*						
Male (%)	7 (33.3%)	16 (36.4%)	21 (65.6%)	44 (45.4%)	**0.019** ^**a**^	
Female (%)	14 (26.4%)	28 (52.8%)	11 (20.8%)	53 (54.6%)		
*School attended*					**0.014** ^**a**^	
None (%)	3 (12.5%)	7 (29.2%)	18 (58.3%)	28 (28.7%)		
College (%)	7 (21.2%)	17 (51.5)	9 (27.3)	33 (34%)		
University (%)	11(30.6%)	19 (52.8%)	6 (20.7%)	36 (37.1%)		
*SOFAS*	Mean (SD)					**(0) vs. (2) 0.004**
	63.86 (2.71)	63.72 (1.87)	52.34 (2.19)	60	**0.000** ^**b**^	**(1) vs. (2) 0.000**
*PID-5*	Mean (SD)					
Detachment	0.90 (0.43)	1.39 (0.52)	1.19 (2.19)	97	**0.003** ^**c**^	**(0) vs. (1) 0.002**
Disinhibition	0.91 (0.31)	1.19 (0.47)	1.24 (0.42)	97	**0.018** ^**c**^	**(0) vs. (1) 0.045** **(0) vs. (1) 0.022**

Bold values significant is set at *p* ≤ 0.05.

a Pearson’s Chi-square test.

b ANOVA.

c MANOVA.

Finally, to investigate common trans-diagnostic personality traits (PID-5) between groups a MANOVA was required. A main effect in the three groups emerged for the PID-5 domains of detachment and disinhibition (Wilks’s Λ = 0.702; *F* = 3.478; *η*_partial_ = 0.162, *p* < 0.001) as shown in Table 3. Negative affectivity and psychoticism were found to be significant on the Levene test and thus excluded from the investigation whereas antagonism was not significant in the Post-Hoc analysis. According to Bonferroni multiple comparison tests, significant differences were confirmed between groups for detachment [*F*(2, 94) = 6.214; *p* = 0.003; *η*_partial_ = 117] and disinhibition [*F*(2, 94) = 4.215; *p* = 0.018; *η*_partial_ = 0.082]. Specifically, the detachment was found to be higher in the group At Risk (M = 1.39) compared to that one Not at risk (M = 0.90) for *p* = 0.003, as well as disinhibition for which the group At Risk (M = 1.19) and Ultra High Risk (M = 1.24) reported higher scores in comparison to the Not at risk group (M = 0.91).

## 4. Discussion

Research on at risk mental states is a crucial field of investigation with implications for early intervention (Carrión et al., 2013; Kraan et al., 2017; van der Gaag et al., 2019; Joa et al., 2021). Studies have intensively focused on predictors of psychotic onset to minimize the likelihood of transition and improve social and occupational functioning.

For this reason, the current study’s first aim was to explore the evolution of the initial diagnosis over 12 months among patients recruited and assigned to Not At risk, At Risk, and Ultra-High-Risk (UHR) groups. One year after the first assessment, the most prevalent category diagnoses were anxiety/depression, personality disorders, and psychosis in the total sample. Specifically, the most common diagnosis in the UHR group was psychosis (40.6%). This result is in line with Yung et al. (2003) who found that 40.8% of the high-risk youths had a psychotic onset after 1 year. One question that is currently unanswered is whether UHR/ARMS conditions – as they are currently conceptualized – should be considered exclusively as a risk condition for transition to psychosis (van Os and Guloksuz, 2017) or as a generic marker of vulnerability to different psychopathologies or even to progression to a generic deterioration in functioning, independent of other clinical disorders in comorbidity. As critically understated by van Os and Guloksuz (2017), perhaps multidimensional psychopathology at baseline in young help-seekers may predict several trajectories, one of which is the so-called transition to psychosis (Albert et al., 2018). Therefore, further studies on this topic, are undoubtedly needed. Moreover, it is possible that the number of transitions would have been greater whether, during the year, UHR patients had not received a psychological – or pharmacological, as required – intervention tailored to their needs (Yung et al., 2003, 2004).

Once the diagnostic categories most associated with high psychotic risk were established, differences in the level of social, occupational functioning, and personality traits were identified. Again, the lowest social and occupational functioning scores were found in the UHR group. Similar findings have been found in previous studies (Yung et al., 2004; Fusar-Poli, 2017; Radua et al., 2018). Specifically, the high-risk population presents a poor quality of life and impairment in social and occupational functioning (Yung and McGorry, 1996; Olvet et al., 2015; Fusar-Poli et al., 2016). These shortcomings are due to negative symptoms, disorganization, metacognitive deficits, and consequently an impaired Theory of Mind (Lin et al., 2012; Carrión et al., 2013; Yung et al., 2015; Kraan et al., 2017). Cotter et al. (2019) highlighted three potential factors that may influence the shortcomings which are (a) exposure to adverse life experiences, (b) the presence of positive and negative symptoms, and (c) cognitive dysfunction. The last two would directly impact social functioning; while having experienced adverse events would negatively affectivity, self-esteem, and self-efficacy, elements in themselves responsible for metacognitive defunctions, and self-stigmatizing attitudes, can reduce social functioning.

Concerning differences in PID-5 personality traits, the highest scores, in detachment and disinhibition, were found in At-risk and UHR groups.

Detachment was found to be a prevalent trait in people with psychotic risk (Meliante et al., 2021). It manifests as avoidance of emotions, social situations, interpersonal withdrawal, and anhedonia. This pattern is consistent with widespread negative symptoms in psychotic risk patients (Freeman and Garety, 2014; Meliante et al., 2021). Moreover, according to research, avoidant behavior (expression of detachment) should be conceived as an attempt to manage positive symptoms by limiting disturbing external stimuli (Pallanti et al., 2000; McMillan et al., 2009). On this line, it is possible to hypothesize that these behaviors are directly involved in poor functioning and unsatisfactory quality of life (Cotter et al., 2019).

Individuals with high disinhibition traits engage in impulsive behaviors without thinking about possible future consequences (Krueger et al., 2012). The high scores, obtained by UHR patients in this dimension, were somehow expected and corroborates previous research (Yung et al., 2019; Hazan et al., 2020). In this regard, conscientiousness (the opposite of disinhibition) has been found to be negatively correlated with psychotic experiences (Shi et al., 2018). Personality traits, perhaps, in part, represent structural tendencies in the affectivity, cognition, and behavior of individuals that might elicit higher levels of stress, contribute to social isolation and reduce opportunities for disconfirmation of psychotic interpretation (Shi et al., 2018). Usually, high levels of disinhibition are common among patients diagnosed with borderline personality disorder (Boldrini et al., 2019; Longenecker et al., 2020). The result obtained in the current study could be explained in different ways. Recent research has focused on identifying the most prevalent personality disorders in high-risk patients, finding BPD as the most prevalent one (Ryan et al., 2017; Boldrini et al., 2019). High scores in disinhibition for UHRs can be explained by appealing to distractibility – one of the facets present in the disinhibition factor. It is known that UHR patients present difficulties in concentration, planning activities, and time management (Cotter et al., 2019), elements highly correlated with high levels of distractibility (Mukwevho, 2018).

To conclude the discussion on personality traits, remarks on negative affectivity, antagonism, and psychoticism are considered appropriate. No statistically significant differences were found between the groups for the traits. Previous studies – which have instead found a significant difference in the levels of negative affectivity – compared groups of patients with psychosis vs. psychiatric patients with different diagnoses, or patients at high psychotic risk vs. healthy controls (Uliaszek et al., 2015; Bastiaens et al., 2019). Thus, probably the lack of significance presented in the current study can be attributed to the type of groups compared. Antagonism is a personality domain not involved in psychotic risk (Fresán et al., 2015; van der Gaag et al., 2019). Regarding psychoticism, the results obtained confirmed those of Drvaric et al. (2018). Indeed, although psychoticism is defined as a set of unusual beliefs and experiences, eccentricity, and perceptual dysregulation (Krueger et al., 2012), no significant differences emerged between the groups. This could mean that psychoticism does not discriminate between psychotic and nonpsychotic patients (Longenecker et al., 2020) and it cannot be considered a trans-diagnostic trait that increases the psychotic risk (Bastiaens et al., 2019).

Although the results obtained can be considered promising, more studies would be needed. In this regard, Boldrini et al. (2020) identified through the SWAP-200 the following prototypical characteristics of patients at high risk for psychosis: avoidance of social relationships, suspiciousness, obsessive thoughts, lack of psychological insight, dysphoric and overwhelming feelings of anxiety and depression, strange and abnormal reasoning processes or perceptual experiences, symptoms of depersonalization and derealization, and negative symptoms of avolition, abulia, blunted affectivity, and impaired role functioning. In addition, as stated by Sevilla-Llewellyn-Jones et al. (2018) it is still not entirely clear to what extent personality may influence the development of psychosis. Personality profiles may not be markers of conversion to psychosis but contribute to high morbidity in individuals with CHR-P.

The study presents different limitations. First, the sample dimension is limited. Second, although the study is longitudinal, only two measures were included. Third, SCID-5 was only administrated during the assessment after 1 year. Moreover, the impact of Covid-19 was not evaluated. Lastly, only a self-report measure (PID-5) was used to assess personality traits. According to the results obtained in previous studies, future research should also include different instruments for personality assessment such as Shedler-Westen Assessment Procedure-200 (SWAP-200) and Millon Multiaxial Inventory (MCMI-III).

## 5. Conclusion

Results obtained by the current study should be considered a first attempt to better understand the diagnostic trajectories and trans-diagnostic personality traits in a group of young help-seekers, specifically in UHR. Findings have two main clinical implications: (a) Psychotherapy should focus specifically on maladaptive traits (b) Treatments for UHR should implement training aimed at improving social and occupational skills. All the traits and impairments identified in this clinical population are disabling during emerging adulthood, a stage of life in which succeeding well socially becomes critical to establishing oneself scholastically, occupationally, and above all, to enjoying satisfying interpersonal relationships (Velthorst et al., 2010).

To improve the clinical utility of psychosis classification systems, it is necessary to consider how symptoms may reflect dimensions of psychopathology that go beyond the boundaries of traditional diagnostic classifications (Longenecker et al., 2020).

## Data availability statement

The raw data supporting the conclusions of this article will be made available by the authors, without undue reservation.

## Ethics statement

The studies involving human participants were reviewed and approved by Niguarda Hospital in Milan’s Ethical Committee (Protocol 305-19052021). The patients/participants provided their written informed consent to participate in this study.

## Author contributions

All authors listed have made a substantial, direct, and intellectual contribution to the work, and approved it for publication.

## Funding

The Catholic University of Sacred Heart financed the publication of this research.

## Conflict of interest

The authors declare that the research was conducted in the absence of any commercial or financial relationships that could be construed as a potential conflict of interest.

## Publisher’s note

All claims expressed in this article are solely those of the authors and do not necessarily represent those of their affiliated organizations, or those of the publisher, the editors and the reviewers. Any product that may be evaluated in this article, or claim that may be made by its manufacturer, is not guaranteed or endorsed by the publisher.
