# Corrigendum: Personality traits and transition to psychosis one year after the first assessment

**DOI:** 10.3389/fpsyg.2023.1170989

**Published:** 2023-05-22

**Authors:** Francesca De Salve, Chiara Rossi, Cesare Cavalera, Lara Malvini, Simona Barbera, Sofia Tagliabue, Mauro Percudani, Osmano Oasi

**Affiliations:** ^1^Department of Psychology, Catholic University of Milan, Milan, Italy; ^2^Department of Mental Health and Addiction Services, Niguarda Hospital, Milan, Italy

**Keywords:** at-risk mental states, ultra high risk, psychosis, personality traits, PID-5, social and occupational functioning, detachment and disinhibition

In the published article, five authors' names were written incorrectly, with first name and surname in the wrong order. Instead of “Malvini Lara, Barbera Simona, Tagliabue Sofia, Percudani Mauro and Oasi Osmano,” the names should be written as “Lara Malvini, Simona Barbera, Sofia Tagliabue, Mauro Percudani and Osmano Oasi.” All relevant parts of the original article have been updated.

Additionally, in the published article, there was an error in the text. “Person correlation” was written instead of “Pearson's Chi-Square Test.”

A correction has been made to **2. Materials and methods**, “*2.4. Statistical analysis*,” paragraph 3. The corrected paragraph is shown below.

“Firstly, Pearson's Chi-Square Test was conducted to investigate the development of psychotic risk in the sample exploring the evolution of the initial diagnosis over 12 months among patients recruited and assigned to three groups (Not at risk, At Risk, Ultra High Risk). Differences in social and occupational functioning (considering SOFAS scores), among the groups, were then explored by conducting a One-way ANOVA. Finally, a MANOVA was used to investigate common trans-diagnostic personality traits (PID-5) of psychotic risk groups. Both ANOVA and MANOVA Bonferroni multiple comparison tests were led. Analyses were performed using SPSS 25.0 statistical software.”

The authors apologize for these errors and state that they do not change the scientific conclusions of the article in any way. The original article has been updated.

